# Multimerization through Pegylation Improves Pharmacokinetic Properties of scFv Fragments of GD2-Specific Antibodies

**DOI:** 10.3390/molecules24213835

**Published:** 2019-10-24

**Authors:** Irina V. Kholodenko, Daniel V. Kalinovsky, Elena V. Svirshchevskaya, Igor I. Doronin, Maria V. Konovalova, Alexey V. Kibardin, Tatyana V. Shamanskaya, Sergey S. Larin, Sergey M. Deyev, Roman V. Kholodenko

**Affiliations:** 1 Orekhovich Institute of Biomedical Chemistry, 10, Pogodinskaya St., Moscow 119121, Russia; irkhol@yandex.ru; 2 Shemyakin-Ovchinnikov Institute of Bioorganic Chemistry, Russian Academy of Sciences, 16/10, Miklukho-Maklaya St., Moscow 117997, Russia; dcalinovschi@yahoo.com (D.V.K.); esvir@mx.ibch.ru (E.V.S.); doroninii@gmail.com (I.I.D.); mariya.v.konovalova@gmail.com (M.V.K.); deyev@mail.ibch.ru (S.M.D.); 3 Real Target LLC, Miklukho-Maklaya St., 16/10, Moscow 117997, Russia; 4 D. Rogachev Federal Research Center of Pediatric Hematology, Oncology and Immunology, 1, Samory Mashela St., Moscow 117997, Russia; alexey.kibardin@gmail.com (A.V.K.); shamanskaya.tatyana@gmail.com (T.V.S.); sergei_larin@mail.ru (S.S.L.); 5 Sechenov First Moscow State Medical University, 8-2, Trubetskaya St., Moscow 119992, Russia

**Keywords:** antibody fragments, pegylation, multimerization, ganglioside GD2, immunotherapy, cancer, neuroblastoma

## Abstract

Antigen-binding fragments of antibodies specific to the tumor-associated ganglioside GD2 are well poised to play a substantial role in modern GD2-targeted cancer therapies, however, rapid elimination from the body and reduced affinity compared to full-length antibodies limit their therapeutic potential. In this study, scFv fragments of GD2-specific antibodies 14.18 were produced in a mammalian expression system that specifically bind to ganglioside GD2, followed by site-directed pegylation to generate mono-, di-, and tetra-scFv fragments. Fractionated pegylated dimers and tetramers of scFv fragments showed significant increase of the binding to GD2 which was not accompanied by cross-reactivity with other gangliosides. Pegylated multimeric di-scFvs and tetra-scFvs exhibited cytotoxic effects in GD2-positive tumor cells, while their circulation time in blood significantly increased compared with monomeric antibody fragments. We also demonstrated a more efficient tumor uptake of the multimers in a syngeneic GD2-positive mouse cancer model. The findings of this study provide the rationale for improving therapeutic characteristics of GD2-specific antibody fragments by multimerization and propose a strategy to generate such molecules. On the basis of multimeric antibody fragments, bispecific antibodies and conjugates with cytotoxic drugs or radioactive isotopes may be developed that will possess improved pharmacokinetic and pharmacodynamic properties.

## 1. Introduction

Ganglioside GD2 represents one of the most attractive and promising targets for cancer immunotherapy. This non-protein molecule belongs to the class of glycosphingolipids and is hyperexpressed primarily in tumors of neuroectodermal origin, whereas its expression on normal non-malignant cells is limited. Ganglioside GD2 is a marker of neuroblastoma, glioma, retinoblastoma, small cell lung cancer, melanoma, and various sarcomas [1,2].

Only one GD2-targeted immunotherapeutic drug, the chimeric monoclonal antibody dinutuximab (tradename Unituxin), is currently approved for clinical practice. It is used as part of combination therapy to treat high-risk neuroblastoma and increases the five-year patient survival by 20% compared to chemotherapy alone. Dinutuximab beta, which is produced in Chinese hamster ovary (CHO) cells as opposed to Sp2/0 mouse myeloma cell line used for production of dinutuximab, has also received marketing approval. Active research is currently being conducted regarding the choice of optimal combination of chemotherapies and Unituxin, as well as the selection of therapy regimens to enhance the efficiency of antitumor activity and reduce side effects in neuroblastoma patients [3].

The antitumor effects of GD2-specific antibodies are based on antibody-dependent cellular cytotoxicity (ADCC) and direct induction of cell death [4]. According to recent data, the contributing role of the complement-dependent cytotoxicity (СDC) in these effects is quite modest, and, moreover, the binding of complement proteins to the antibody Fc fragment is responsible for one of the main side effects associated with the use of GD2-specific monoclonal antibodies, namely antibody-induced allodynia [5].

The antigen-binding fragments of GD2-specific antibodies lack the Fc fragment, and hence the associated side effects. These antibody fragments also demonstrate better penetration into solid tumors and present a significant potential for therapy, molecular imaging, and cancer diagnostics [6,7]. At the same time, monomeric antigen-binding antibody fragments have therapeutic limitations associated with their rapid elimination from the body, reduced affinity for the antigen, and low stability in solution compared to full-length antibodies [6].

Pegylation is one of the well-developed strategies used to improve pharmacokinetic and pharmacodynamic properties of antibody fragments [8,9,10]. Pegylation increases the hydrodynamic radius of the molecule and, thus, reduces its renal clearance and increases its half-life in the blood. Pegylated proteins are also absorbed and accumulated faster by the tumor mass compared to non-modified proteins [11,12,13].

Pegylation may be used to oligomerize antibody fragments [14], which, as a rule, leads to multivalent interaction of the resulting constructs with the target antigen. Such interaction not only increases affinity due to the avidity effect but it can also improve functional properties of the molecules and compensate for the absence of Fc-mediated immune functions characteristic for full-length antibodies. Therefore, development of multimeric pegylated fragments of GD2-specific antibodies has a considerable potential for treatment and diagnostics of cancer. In this study, we generated pegylated monomers and multimers of scFv fragments of GD2-specific antibodies using maleimide-thiol chemistry, evaluated their antigen-binding properties, and assessed their cytotoxic effects in GD2-positive tumor cell lines. We focused specifically on the study of the circulation time of pegylated antibody fragments in the blood of mice and on the tumor uptake of the molecules in a syngeneic GD2-positive mouse cancer model.

## 2. Results

### 2.1. Generation of Pegylated scFv Fragments

#### 2.1.1. Design and Expression of the scFv Fragment

**Figure 1:**
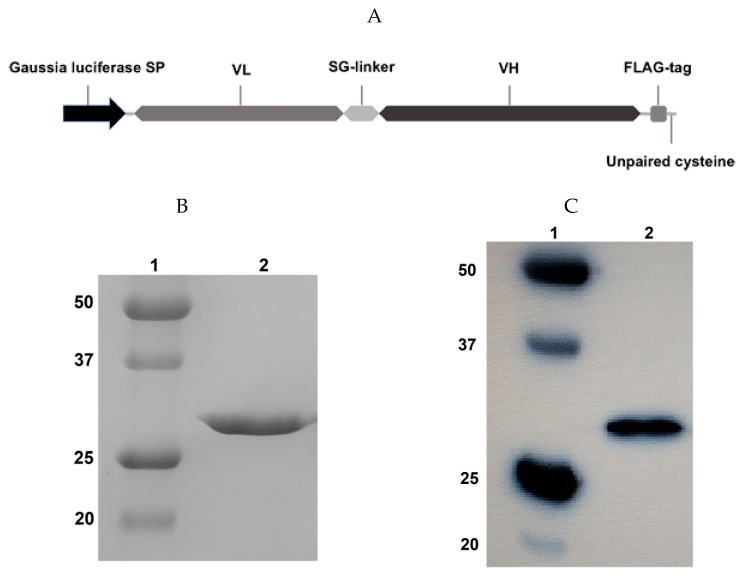
Design and expression of the scFv fragment 14.18. (**A**) Structure of the scFv fragment 14.18. (**B**) 12% reducing sodium dodecyl sulfate polyacrylamide gel electrophoresis (SDS-PAGE); 1, molecular weight protein markers; 2, scFv fragment 14.18. (**C**) Western blot analysis; the membrane was incubated with HRP-labelled anti-FLAG antibodies (1:6000). Visualization with 1-Step Ultra TMB-Blotting Solution.

We designed the scFv antibody fragment based on the sequence of the variable domains of the GD2-specific antibody 14.18 reported by Bolesta et al. [15]. The scFv fragment sequence was arranged in the VL-VH orientation, and the commonly used (G_3_S)_4_ linker, a FLAG-tag for detection (amino acid sequence motif DYKDDDDK), and a C-terminal free cysteine for subsequent site-directed pegylation were all introduced into the antibody framework (Figure 1A).

Following transient gene expression in Expi293F cells and purification by protein L chromatography, we analyzed the resultant protein fraction by 12% reducing polyacrylamide gel electrophoresis (Figure 1B) and immunoblotting using horseradish peroxidase (HRP)-labelled anti-FLAG antibodies (Figure 1C). One protein band with a 27 kDa molecular weight was observed, which corresponds to the expected molecular weight of the scFv fragment 14.18. The resulting protein also effectively binds with anti-FLAG antibodies (Figure 1C), which confirms the presence of the FLAG-tag in the construct. After purification, the secreted protein was present predominantly in monomeric form, despite the fact that the molecule contained a C-terminal free cysteine. When an identical scFv was expressed in *E. coli*, it formed water-insoluble dimers due to generation of a disulfide bridge with the C-terminal free cysteine of another scFv molecule. It is likely that partial endogenous cysteine capping modification of scFv fragments described by Zhong et al. [16] occurred in the eukaryotic expression system, which prevented the formation of inactive dimers. At the same time, in order to reliably prevent possible dimerization of fragments by free terminal cysteines, we also performed exogenous cysteine capping by adding oxidized glutathione to the culture medium. The transfection efficiency was approx. 20 mg/L, and the stability of the isolated protein was higher compared with the scFv fragments of GD2-specific antibodies which we had previously obtained in *E. coli* [17].

#### 2.1.2. Production of Antibody Fragment Conjugates

**Figure 2:**
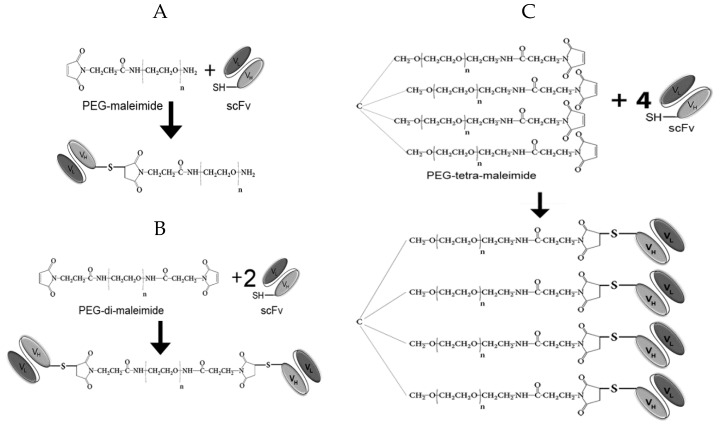
Schematic representation of pegylation reactions. (**A**) Generation of scFv-PEG conjugates using PEG-maleimide. (**B**) Generation of di-scFv-PEG multimers using PEG-di-maleimide. (**C**) Generation of tetra-scFv-PEG multimers using PEG-tetra-maleimide.

It is known from literature that antigen-binding properties, circulation time in blood, as well as tumor uptake depend on polyethylene glycol (PEG) molecule size to a significant extent [11]. Even a slight difference in PEG size may lead to a profound increase of the hydrodynamic radius of the modified molecule and to a change in the above-mentioned properties. Therefore, it appeared important to assess the impact of PEG size on the properties of the pegylated monomeric and multimeric antibody fragments. Regarding the PEG molecule choice for generation of multimeric fragments, it is necessary to take into consideration the total molecular weight of the pegylated multimers which has to be in the range of 60–150 kDa to avoid renal clearance, as well as not to exceed the molecular weight of full-length antibodies. Based on our tasks and on commercially available reagents, we employed several variants of PEG-maleimide molecules in the experimental setup, namely PEG-maleimide 5 kDa, PEG-maleimide 10 kDa, PEG-maleimide 40 kDa, PEG-di-maleimide 2 kDa, PEG-di-maleimide 5 kDa, and PEG-tetra-maleimide 10 kDa (all from JenKem Technology, USA). The unpaired cysteine introduced to the C-terminal of the scFv 14.18 sequence was used for site-directed pegylation with maleimide-activated polyethylene glycol derivatives. Pegylation reaction schemes are presented in Figure 2.

**Figure 3:**
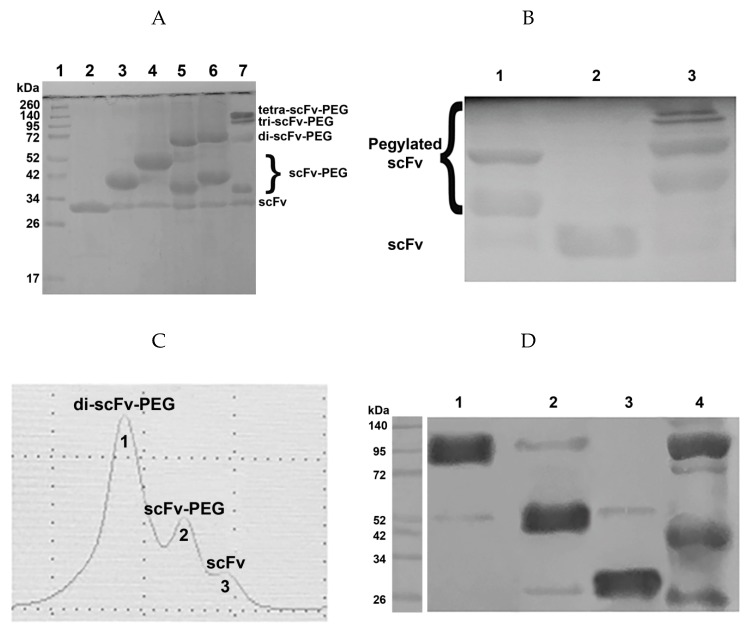
Efficiency of the pegylation reaction. (**A**) 12% SDS-PAGE of the pegylation reaction products; 1, molecular weight protein markers; 2, intact scFv fragments 14.18; 3, pegylation with PEG-maleimide 5 kDa; 4, pegylation with PEG-maleimide 10 kDa; 5, pegylation with PEG-di-maleimide 2 kDa; 6, pegylation with PEG-di-maleimide 5 kDa; 7, pegylation with PEG-tetra-maleimide 10 kDa. (**B**) Western blot of the pegylation reaction products; membrane was incubated with HRP-labelled anti-FLAG antibodies (1:6000); 1, pegylation with PEG-di-maleimide 5 kD; 2, intact scFv fragments 14.18; 3, pegylation with PEG-tetra-maleimide 10 kD. (**C**) size-exclusion chromatographic purification (Superdex 200 10/300 GL column), pegylation reaction with PEG-di-maleimide 5 kD. (**D**) western blot following size-exclusion chromatographic purification, pegylation reaction with PEG-di-maleimide 5 kD; 1, fraction of pegylated di-scFv fragments; 2, fraction of pegylated mono-scFv fragments; 3, fraction of non-pegylated scFv fragments; 4, total protein mixture after pegylation with PEG-di-maleimide 5 kD.

To generate the constructs pictured in Figure 2, we initially carried out mild reduction of the antibody fragments with TCEP, followed by conjugation with maleimide-activated derivatives of polyethylene glycol in various molar ratios. The pegylation efficiency was analyzed based on the results of sodium dodecyl sulfate polyacrylamide gel electrophoresis (SDS-PAGE) and subsequent Western blotting with detection of the FLAG-tag introduced into the scFv fragment (Figure 3A,B).

Normalized values from quantitative densitometry analysis of western blot bands for (Figure 3B,D) can be found in Figures S1 and S2, respectively (see Supplementary materials).

For each pegylation reaction of the scFv fragment with the corresponding maleimide-activated polyethylene glycol variant, the molar ratio of the components and the reaction time were optimized. The optimal protein:PEG ratios for PEG-maleimide, PEG-di-maleimide, and PEG-tetra-maleimide in the given reaction conditions (see Materials and Methods) are 1:10, 1:2, and 1:1, respectively. After optimization of the pegylation conditions, the reaction efficiency constituted at least 80% (Figure 3A). Proteins conjugated with high molecular weight PEG molecules are characterized by anomalous mobility in polyacrylamide gel compared to unmodified proteins, which was most noticeable for PEG-di-maleimide 40 kDa (data not shown). In the case of protein conjugation with lower molecular weight PEG molecules, the reaction products corresponded to the calculated (expected) molecular weights of pegylated mono-, di-, and tetra-scFv fragments (Figure 3A).

For additional purification of the resulting products from non-bound maleimide-activated polyethylene glycol and separation of the reaction products in case of pegylation with PEG-di- and PEG-tetra-maleimide, size-exclusion chromatography was performed on a Superdex 200 10/300 GL column (Beckman System Gold high-performance liquid chromatography (HPLC) system, see Materials and methods). Chromatographic separation resulted in individual fractions, which were collected and assigned to correspondent protein-PEG conjugates by western blot analysis. As an example, (Figure 3C,D) show size-exclusion chromatographic purification of scFv fragments 14.18 pegylated with PEG-di-maleimide.

### 2.2. Antigen-Binding Properties of Modified scFv Fragments of GD2-Specific Antibodies

ScFv fragments are characterized by monovalent interaction with the antigen, in contrast to the bivalent binding of full-length antibodies. If the antigens are proteins or peptides, monovalent binding usually does not significantly reduce the interaction strength, and affinity can be maintained in a low nanomolar range. In the case of carbohydrate antigens, the interaction decreases significantly and the dissociation constants can exceed 10 μM [18]. In addition, modification of the fragments through pegylation can affect binding to the antigen on its own. Therefore, an important step within our experimental setup was the evaluation of the interaction of the modified scFv fragments with ganglioside GD2 by enzyme-linked immunosorbent assay (ELISA), as well as with GD2-positive tumor cells by flow cytometry.

**Figure 4:**
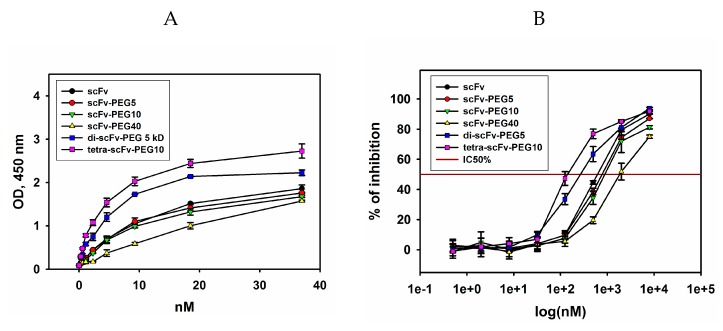
Binding of intact and modified scFv fragments of GD2-specific antibodies with ganglioside GD2. (**A**) direct enzyme-linked immunosorbent assay (ELISA); ganglioside GD2 was adsorbed on the plate, serial dilutions of scFv fragments were added to the wells; after incubation with HRP-labelled anti-FLAG antibodies (1: 6000), the reaction was developed by ultra TMB-ELISA substrate solution. (**B**) competitive ELISA; GD2 was adsorbed on the plate, a mix of biotinylated GD2-specific antibodies 14G2a at a concentration of 50% saturation and different concentrations of scFv fragments was added to the wells; after incubation with HRP-labelled streptavidin (1:6000), the reaction was developed by Ultra TMB-ELISA Substrate Solution. Data are represented as mean ± SEM.

The binding of monomers and multimers of pegylated scFv fragments 14.18 to ganglioside GD2 in direct ELISA is represented in Figure 4A. All modified antibody fragments interacted with GD2, however, the binding efficiency was different. The highest antigen-binding efficiency was observed for di- and tetra-scFv fragments of GD2-specific antibodies. Moreover, tetramers of scFv fragments exhibited higher binding to GD2 than dimers. Dimers generated using PEG-di-maleimide 2 kDa and PEG-di-maleimide 5 kDa demonstrated similar antigen-binding properties (data not shown). Monomeric intact antibody fragments interacted with ganglioside GD2 weaker than di- and tetra-scFv fragments, and pegylation led to a slight decrease in binding when 5-kDa and 10-kDa PEG-maleimide molecules were used instead of the original scFv fragment. When 40-kDa PEG-maleimide was conjugated to the scFv fragment, a decrease in the interaction with GD2 was observed compared to both the original scFv and multimeric scFv fragments.

In order to confirm the data obtained in direct ELISA and to assess the IC50% values, competitive ELISA was carried out. IC50% was calculated as the respective scFv fragment conjugate concentration at which 50% inhibition of the binding of full-length GD2-specific antibodies to ganglioside GD2 takes place during competitive interaction. For carrying out competitive ELISA, biotinylated GD2-specific antibodies 14G2a at a concentration of 50% saturation (half maximal effective concentration (EC50%)), which was 0.33 nM (data not shown), were mixed and incubated with serial dilutions of scFv fragments. After incubation with streptavidin-HRP and reaction development, IC50% was determined for each variant of scFv fragment conjugates of GD2-specific antibodies (Figure 4B). The results obtained in the competitive ELISA confirmed the patterns found in direct ELISA. The modified scFv fragments differed in their ability to inhibit the interaction of full-length antibodies with ganglioside GD2. The minimal IC50% (half maximal inhibitory concentration) values were observed for pegylated di- and tetra-scFv fragments and constituted 143 ± 8 nM and 262 ± 11 nM, respectively. The IC50% values for monomeric intact and pegylated antibody fragments were significantly higher and constituted 620 ± 14 nM, 776 ± 20 nM, and 880 ± 17 nM for intact scFv fragments, scFv-PEG 5 kDa, and scFv-PEG 10 kDa, respectively. IC50% for scFv fragments pegylated with PEG-maleimide 40 kDa was even higher, in the micromolar range. It is likely that in this case the PEG molecule with large molecular weight and branched structure partially masked the interaction of the antibody fragment with ganglioside GD2. The obtained data demonstrate that pegylation of intact scFv fragments with monovalent PEG-maleimides of increasing molecular weight leads to a non-linear decrease in the binding with the antigen, whereas multimerization of the scFv fragments by polyvalent PEG-maleimides substantially increases this binding.

**Figure 5:**
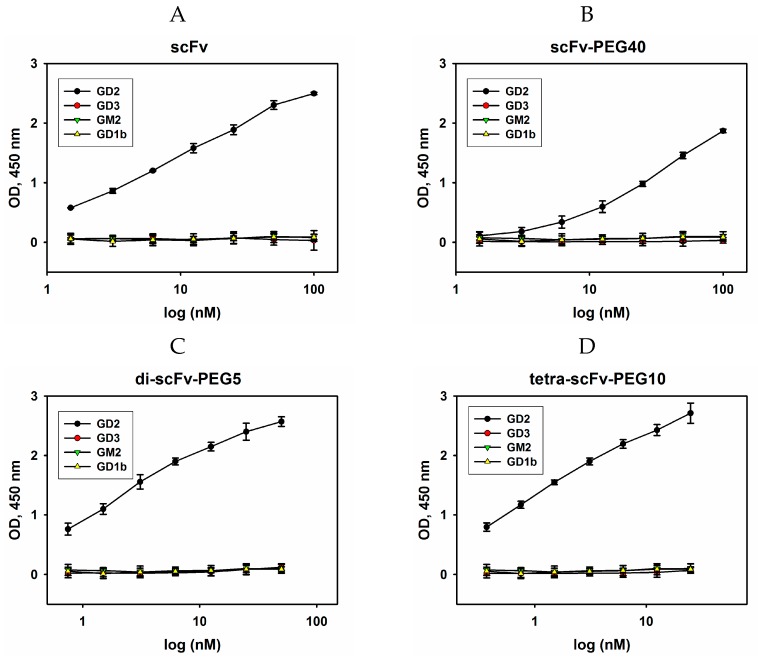
Evaluation of cross-reactivity of intact and modified scFv fragments in direct ELISA. GD2, GD3, GM2, and GD1b gangliosides were adsorbed on the plate; serial dilutions of intact scFv fragments (**A**), scFv-PEG 40 kDa (**B**), di-scFv-PEG 5 kDa (**C**), and tetra-scFv-PEG 10 kDa (**D**) were added to the wells. After incubation with HRP-labelled anti-FLAG antibodies (1:6000), the reaction was developed by Ultra TMB-ELISA Substrate Solution. Data are represented as mean ± SEM.

In some cases, an increase in the affinity of GD2-specific antibodies as well as in the affinity of their antigen-binding fragments due to affinity maturation or predicted point mutations leads to cross-reactivity with other gangliosides that are structurally similar to ganglioside GD2. Zhao et al. [19] showed a significant increase in the affinity of a number of scFv fragments of GD2-specific antibodies hu3F8 selected from the yeast display of randomized Fv mutations. The scFv fragments obtained were characterized by a considerable increase of the dissociation constant (Kd increased by 10–50 times), and at the same time cross-reactivity with other gangliosides appeared, primarily with ganglioside GD1b which is not a tumor-associated molecule. It is likely that the E101K mutation within the antigen-binding domain of the GD2-specific antibody 14G2a that leads to an increase in binding to ganglioside GD2 [20], also influences cross-reactivity and, as a result, causes significant side effects in mouse tumor models when modified GD2-binding domains are employed in сhimeric antigen receptor (CAR) designs [21,22]. Therefore, it seemed reasonable for us to evaluate the possible appearance of cross-reactivity of the modified scFv fragments of GD2-specific antibodies towards other gangliosides. Structurally, GD3, GM2, and GD1b gangliosides are the closest to GD2; therefore, the possible binding of the modified scFv fragments with those gangliosides was assessed in direct ELISA (Figure 5A).

For all variants of GD2-specific scFv fragment constructs, no cross-reactivity with GD3, GM2, and GD1b gangliosides was observed in the entire range of selected concentrations, and cross-reactivity content was below 1% even at high concentrations.

**Figure 6:**
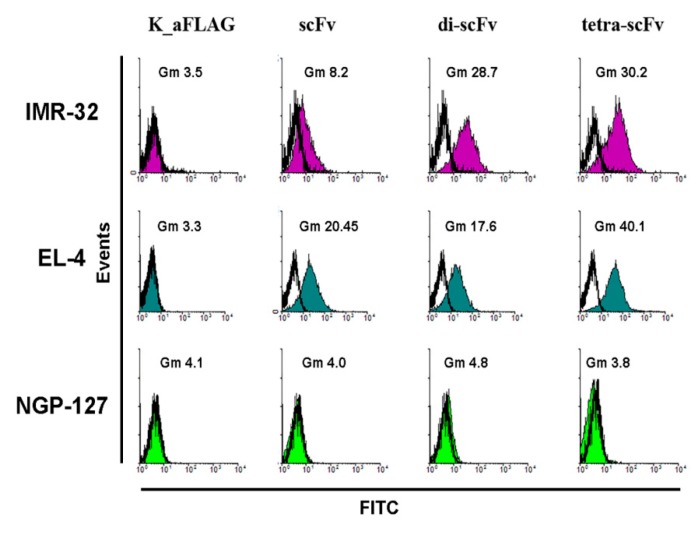
Flow cytometry analysis. Incubation of IMR-32, EL-4, and NGP-127 cell lines with intact scFv fragments, di-scFv-PEG, and tetra-scFv-PEG, followed by staining with fluorescein-5-isothiocyanate (FITC)-labelled anti-FLAG antibodies (1:500). K_aFLAG—Control cells stained only with FITC-labelled anti-FLAG antibodies.

We next used flow cytometry to analyze the binding of multimeric scFv fragments with ganglioside GD2 on the cell surface of several GD2-positive and GD2-negative cell lines. All the molecules, including intact scFv fragments, di-scFvs, and tetra-scFvs, were efficiently bound on the cell surface of GD2-positive IMR-32 and EL-4 cell lines, but not GD2-negative NGP-127 cell line (Figure 6).

Thus, we show that the resulting scFv fragment constructs are capable of not only binding to the pure antigen adsorbed on an ELISA plate, but also to tumor cells expressing ganglioside GD2 on the cell surface. On top of this, the flow cytometry analysis supplements the results of the ELISA, indicating that multimerization of scFv fragments 14.18 does not result in cross-reactivity.

### 2.3. Сytotoxic Effects of Modified scFv Fragments of GD2-Specific Antibodies

**Figure 7:**
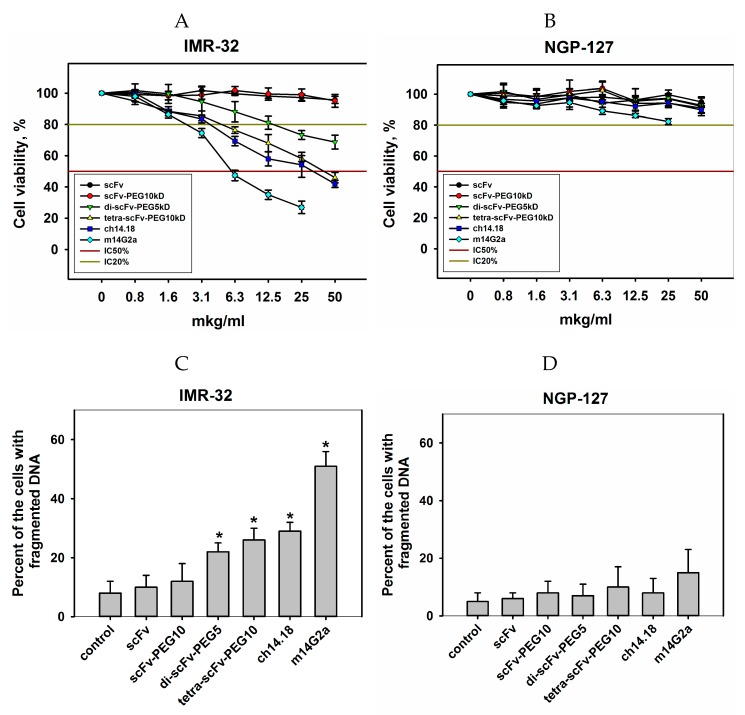
Direct cell death effects following incubation with intact scFv fragments, pegylated monomers of scFv fragments, pegylated di- and tetra-scFv fragments, and full-length GD2-specific antibodies (chimeric 14.18 and mouse 14G2a). (**A**) GD2-positive IMR-32 cell line, 3-[[4,5]-dimethylthiazol-2-yl]-2,5-diphenyltetrazolium bromide (MTT)-assay after 72 h incubation; (**B**) GD2-negative NGP-127 cell line, MTT-assay after 72 h incubation; (**C**) IMR-32 cell line, propidium iodide (PI)-test after 24 h incubation; (**D**) NGP-127 cell line, PI-test after 24 h incubation. Control cells incubated with phosphate-buffered saline (PBS). Data are represented as mean ± standard error of the mean (SEM). *—Indicates a value that significantly differs from the control value at *p* < 0.05 (Student’s *t*-test, *n* = 5).

A number of works have provided evidence that different GD2-specific antibodies are able to induce direct cell death in GD2-positive cells [23,24,25]. In this regard, it was particularly interesting for us to evaluate and compare the direct cytotoxic effects of intact and modified scFv fragments of GD2-specific antibodies. The effects of the antibody fragment conjugates on the viability of GD2-positive IMR-32 and GD2-negative NGP-127 human neuroblastoma cell lines were evaluated in MTT (3-[[4,5]-dimethylthiazol-2-yl]-2,5-diphenyltetrazolium bromide) and propidium iodide (PI) assays (Figure 7).

In both assays, it was shown for the GD2-negative NGP-127 cell line that both intact GD2-specific scFvs, all pegylated conjugates, and full-length GD2-specific antibodies did not inhibit proliferation and did not increase the number of cells with fragmented DNA at all selected concentrations. In the case of the GD2-positive IMR-32 cell line, intact and pegylated monomeric scFv fragments did not manifest pronounced cytotoxic effects even at high doses, however, in contrast to monomeric scFv fragments, pegylated di- and tetra-scFv fragments reduced the viability of IMR-32 cells and increased the number of cells with fragmented DNA. Tetramers of scFv fragments induced cytotoxic effects comparable to the effects of full-length chimeric GD2-specific antibodies. For the other GD2-positive cell line EL-4, the same pattern was observed (Figure S3А,B). Additional experiments are required to study the mechanisms of cell death induced by multimeric fragments of GD2-specific antibodies, as well as to identify common and distinct features of the processes triggered by scFv fragments and full-length GD2-specific antibodies. Regardless of the nature of these effects, induction of direct cell death will likely serve as an additional antitumor mechanism for novel anti-GD2 bispecific antibodies and conjugates with drugs or radioisotopes.

### 2.4. Circulation Time in Blood and Tumor Uptake

#### 2.4.1. Fluorescent Labelling of Modified scFv Fragments with Sulfo-Cyanine5

One of the main reasons for pegylation of the recombinant scFv fragments of GD2-specific antibodies was to increase their circulation time in the blood that is very short for non-modified scFv fragments [6]. In order to analyze the circulation time of the pegylated scFv fragments in the blood, we labelled them with the fluorescent dye Sulfo-Cy5, which allows to monitor the fluorescence intensity and hence the conjugate concentration in the blood serum of mice at different time points. The choice of this near-infrared dye was based on its long-wavelength emission maximum (662 nm), which makes it possible to avoid a significant contribution of autofluorescence of tissues and organs to the assay results [26].

**Figure 8:**
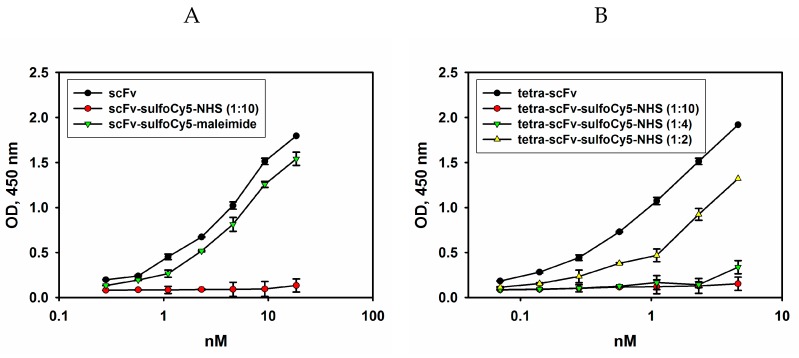
Direct ELISA. Ganglioside GD2 was adsorbed on the plate; serial dilutions of scFv fragments labelled with Sulfo-Cy5-NHS (protein:dye ratio = 1:10) or Sulfo-Cy5-maleimide (**A**), or serial dilutions of tetra-scFv fragments labelled with Sulfo-Cy5-NHS (protein:dye ratios = 1:10, 1:4, or 1:2) (**B**) were added to the wells. After incubation with HRP-labelled anti-FLAG antibodies (1:6000), the reaction was developed by Ultra TMB-ELISA Substrate Solution. Data are represented as mean ± SEM.

Initially, the conjugation of pegylated scFv fragments with Sulfo-Cyanine5 activated ester (Sulfo-Cy5-NHS) was carried out according to the manufacturer’s protocol. Following the pegylation reaction and purification from unreacted dye, the antigen-binding properties of Cy5-labelled original and pegylated scFv fragments were evaluated by direct ELISA. In Figure 8, comparison of the binding of the intact scFv fragments, fluorescently labelled scFv fragments and pegylated tetra-scFv fragments to ganglioside GD2 is provided.

As shown in Figure 8, the use of high concentrations of Sulfo-Cy5-NHS dye resulted in the loss of binding of non-pegylated scFv and tetra-scFv fragments to ganglioside GD2, and the same patterns were characteristic of other pegylated fragments (data not shown). For this reason, the ratios of Sulfo-Cy5-NHS dye and pegylated scFv fragments introduced into the reaction were optimized in such a way as to retain approximately 80% of antigen-binding activity of the non-labelled scFv-PEG conjugates (Figure 8B). At the same time, the original non-pegylated scFv fragments contain the C-terminal free cysteine, which was employed to attach a different dye, Sulfo-Cyanine5-maleimide, by the maleimide-thiol reaction. This approach made it possible to almost completely retain the antigen-binding activity of the original scFv fragments (see Figure 8A). Sulfo-Cyanine5-maleimide was also used to generate Cy5-labelled GD2-specific full-length antibodies 14.18 (data not shown). The molar ratio of the Sulfo-Cyanine5-maleimide dye and the scFv fragment (or full-length antibody) within the conjugate under the optimized conditions was 0.65. For each variant of pegylated scFv fragments, except for scFv-PEG 40 kDa, the corresponding dye:protein ratios within the conjugate were determined. Optimized dye to protein ratios did not significantly affect binding to the antigen and were in the range of 0.63–0.68 dye molecules per scFv fragment.

#### 2.4.2. Analysis of Circulation Time and Tumor Uptake of Intact and Modified scFv Fragments

**Figure 9:**
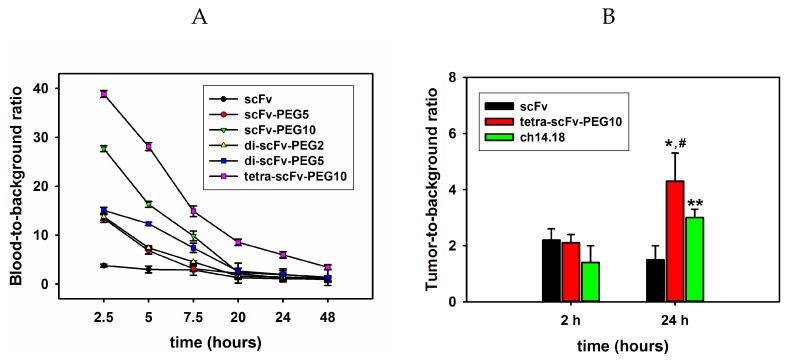
Circulation time and tumor uptake of intact and pegylated scFv fragments after intravenous injection. (**A**) The dynamics of concentration decrease of intravenously administered Cy5-labelled original and modified scFv fragments of GD2-specific antibodies in the blood of mice. (**B**) Accumulation of Cy5-labelled intact scFv, pegylated tetra-scFv fragments, and full-length GD2-specific ch14.18 antibodies in the tumor. Data are represented as mean ± SEM. *—Indicates a value that significantly differs in scFv and tetra-scFv groups at *p* < 0.05 (Student’s *t*-test, *n* = 5), *—Indicates a value that significantly differs in scFv and antibodies groups at *p* < 0.05 (Student’s *t*-test, *n* = 5), #—Indicates a value that significantly differs in tetra-scFv groups and antibodies groups at *±* < 0.05 (Student’s *t*-test, *n* = 5).

For evaluating the circulation time of the molecules in the blood, fluorescently labelled scFv fragment conjugates (150 μg/mouse or 7.5 mg/kg) were intravenously injected into C57BL/6 mice. At certain time points, aliquots of blood were collected, and fluorescence was evaluated for each type of the injected molecule. Figure 9A shows the ratios of the mean fluorescence intensity of Sulfo-Cy5-labelled scFv fragment conjugates in blood plasma to the mean fluorescence intensity of blood plasma of intact mice (blood-to-background (B-T-B) ratio) over time. It can be seen that already at 2.5 h post-injection, a small amount of monomeric non-pegylated scFv fragments remains in the blood and the B-T-B ratio is less than 4. Pegylation considerably increases the circulation time of the scFv fragments in the blood; moreover, circulation time directly depends on the molecular weight of the attached PEG molecule. For example, 2.5 h post-injection, the pegylated monomer scFv-PEG 10 kDa had a B-T-B ratio two times higher than that for scFv-PEG 5 kDa. Similarly, 5 h post-injection, the B-T-B ratio for di-scFv-PEG 5 kDa was 1.7 times higher than for di-scFv-PEG 2 kDa. Multimerization of the fragments further increases the circulation time. While the difference in circulation time for scFv-PEG 5 kDa and di-scFv-PEG 5 kDa was not very significant, and the biggest difference in the B-T-B ratio between mono-scFv and di-scFv fragments was observed 5 and 7 h post-injection (12.3211 ± 0.3 vs. 6.8196 ± 0.7 and 7.3907 ± 0.9 vs. 3.1314 ± 0.6, respectively), the circulation time of tetra-scFv-PEG 10 kDa was significantly higher than that of scFv-PEG 10 kDa, which was observed for B-T-B ratios at all time points. Moreover, a sufficiently high concentration of pegylated tetra-scFv fragments in the blood was maintained even 48 h post-injection; the B-T-B ratio was 3.4297 ± 0.5 for the tetra-scFv-PEG 10 kDa vs. 1.2621 ± 0.17 for the scFv-PEG 10 kDa. Thus, both pegylation and multimerization substantially increase the circulation time of scFv fragments in the blood, and the increase in circulation time directly correlates with the molecular weight of PEG (Figure 9A). This is probably the result of the decrease of renal clearance of the constructs. In preliminary biodistribution experiments, we saw a decrease in the accumulation of tetrameric scFv in the kidneys relative to monomeric scFv by approximately 2.5–3 times and a slight increase in liver accumulation

On the next stage of the experiment, an EL-4 syngeneic tumor model was created to compare the ability of the intact and modified multimeric scFv fragments and full-length GD2-specific antibodies ch14.18 to accumulate in the tumor (Figure 9B). Sulfo-Cy5-labelled non-pegylated scFv, pegylated tetra-scFv fragments, and GD2-specific antibodies ch14.18 were intravenously injected into mice in which the tumor size reached a volume of approx. 300 mm^3^. Mice were sacrificed 2 or 24 h post-injection, tumors were removed, and tumor extracts normalized by weight were obtained. For all molecules at both time points an increase in fluorescence intensity was documented compared with the control samples (extracts obtained from tumors of mice that did not receive Sulfo-Cy5-labelled antibodies or fragments). Two hours after the injection of the molecules, the ratio of fluorescence intensity to the background (tumor-to-background (T-T-B) ratio) was the same for scFv and tetra-scFv fragments, and was equal to two. For full-length antibodies this ratio was 1.4 ± 0.6. At the same time, the accumulation of tetra-scFv fragments in the tumor 24 h after the injection was significantly higher than that of intact scFv fragments; the T-T-B ratio for scFv and tetra-scFv fragments was 1.5 ± 0.9 and 4.3 ± 1.2, respectively. T-T-B ratio for tetra-scFv fragments was also higher than that for full-length antibodies, which was 3 ± 1.4 (Figure 9B).

## 3. Discussion

GD2-directed immunotherapy has been actively developing in recent years, and dinutuximab has become an integral component of multimodal therapy for high-risk neuroblastoma, significantly improving event-free survival (EFS) and overall survival (OS) in patients [3]. Extensive research is being conducted to select optimal regimens, modes of administration, and combination therapies to enhance the efficiency of dinutuximab; so, for example, clinical trials employing dinutuximab in combination with irinotecan and temozolomide have provided encouraging results, while prolonged intravenous infusion of the drug reduces side effects characteristic of the standard short-term infusion procedure [3,27,28]. At the same time, full-length GD2-specific antibodies carry inherent limitations that are unlikely to be completely eliminated without changing the format of the molecule. Due to their size, full-length IgGs have a limited ability to penetrate and accumulate in solid tumors, which is the main reason for reduced efficiency of antibody therapy of solid tumors in general and GD2-directed therapy in particular [29].

The properties of ganglioside GD2 as a tumor-associated marker in the context of neuroblastoma are very promising since more than 95% of neuroblastoma cases are GD2-positive and are characterized by a high level of expression of this antigen [30], and also, the ganglioside-antibody complex is highly stable [31]. Thus, the efficacy of dinutuximab in patients cannot be considered optimal.

A different yet a very significant limitation of GD2-specific full-length antibodies is neuropathic pain caused by their interaction with peripheral sensory neurons. Such on-target/off-tumor toxicity accompanies most cases of the use of dinutuximab in patients. Developing Fc-engineered GD2-specific antibodies with point mutations introduced to reduce the binding of the complement system as the main mechanism responsible for pain only partially alleviates this side effect. A clinical trial of the humanized GD2-specific antibody hu14.18K322A designed to reduce complement activation still identified pain as one of the main side effects [32]. At the same time, that side effect was absent in preclinical trials of GD2-specific CAR T cells and bifunctional antibody fragments that only contained GD2-binding fragments [33]. Anti-GD2 CARTs and anti-GD2/CD3 scFv-based bispecific antibodies are among the most promising GD2-directed next-generation immunotherapeutics. Efficacy of GD2-specific CAR T cells was demonstrated not only for neuroblastoma but also for other GD2-positive types of tumors, specifically, for glioma [34]. However, the use of antigen-binding fragments of GD2-specific antibodies is not restricted to development of bispecific antibodies and incorporation into chimeric antigen receptors. Similarly to antibody fragments specific to other tumor markers, they also represent a framework for generation of antibody-based therapeutics such as immunotoxins, immunocytokines, radioimmunotherapeutics, and antibody-drug conjugates [35,36,37]. These approaches represent potent strategies to compensate for the absence of Fc-mediated effector functions in the antigen-binding fragments [6]. At the same time, an important obstacle that may limit the antitumor potential of antigen-binding antibody fragments lies in their rapid removal from the body and reduced affinity of interaction with the antigen compared with full-length IgGs [6].

In this study, we compare antigen-binding properties, direct cytotoxic effects, circulation time, and tumor uptake of monomeric scFv fragments and pegylated multimeric scFv fragments of GD2-specific antibodies. We have produced scFv fragments 14.18 in an eukaryotic expression system and have confirmed that they specifically bind to ganglioside GD2. At the same time, the strength of this interaction was significantly lower than that for the corresponding full-length antibodies. Optimization of the sequence of the antibody fragment may slightly increase the binding, however, the monovalent character of the interaction between the scFv fragment and the antigen, which is generally weak per se for carbohydrate antigens [18], seems to be the main reason for the decrease of the binding strength compared with bivalent IgGs. To generate pegylated scFv fragments, we conducted a site-directed pegylation reaction via the C-terminal free cysteine with mono-, bi-, or tetrafunctional PEG-maleimide molecules. As a result of the optimization of reaction conditions, 5-kDa- and 10-kDa-pegylated monovalent scFv fragments characterized by minimal loss of antigen-binding properties compared to naked scFvs were produced, whereas pegylation with the 40 kDa PEG molecule led to a significantly reduced interaction of the pegylated fragment with ganglioside GD2. For fractionated di-scFv and tetra-scFv fragments characterized respectively by bi- or tetravalent interaction with the antigen, a significant increase of the binding to GD2 which was not accompanied by the appearance of cross-reactivity with other gangliosides was observed. Unlike monomeric scFvs, multimeric di-scFv and tetra-scFv antibody fragments exhibit objective direct cytotoxic effects in GD2-positive tumor cells, as evaluated by reduction of cell viability and increase of cell count with fragmented DNA. At the same time, these effects are less pronounced compared to the effects of the corresponding full-length antibodies. Multimerization and pegylation significantly increase circulation time of the monomeric scFv fragments in the blood. We also show in a syngeneic GD2-positive mouse cancer model that multimerization through pegylation positively affects the tumor uptake of the molecules.

## 4. Materials and Methods

### 4.1. Expression and Purification of scFv

ScFv fragments of GD2-specific antibodies 14.18 (scFv fragments 14.18) were produced using transient gene expression in mammalian cells (Expi293 Expression system, Thermo Fisher Scientific Waltham, MA, USA). For this, sequences of the variable regions of the light and heavy chains of the 14.18 antibody in the VL-VH orientation were codon-optimized for maximal expression in mammalian cells (synthesized by GenScript Piscataway, NJ, USA) and cloned between the XbaI and AgeI restriction sites of the eukaryotic expression vector pcDNA3.4. The identity of the construct was confirmed by analytical restriction digests. The vector was then purified by QIAGEN Plasmid Maxi Kit (QIAGEN GmbH, Hilden, Germany) according to the manufacturer’s instructions. Prior to transfection, the specimens were sterilized by filtration through a 0.22 μm membrane. Transfection was performed in 30 mL culture volume, following the protocol for the expression system. Cell culture aliquots were taken 2–4, and 5 days post-transfection to calculate viable cell density and percent viability and to determine target protein content in the medium by SDS-PAGE and immunoblotting. The antibody-expressing positive control vector included in the expression system kit was used as a positive control for transfection under recommended conditions; the expression levels of the control antibody corresponded to the expected values. ScFv fragments 14.18 were isolated from the cell culture supernatants by protein L chromatography (HiTrap Protein L purification columns, GE Healthcare, Chicago, IL, USA). The columns were equilibrated with binding buffer, followed by the loading of the supernatants containing scFv fragments to the resin and wash steps to discard unbound protein. ScFv fragments were eluted with pH 3.0 elution buffer, and the resultant solutions were immediately neutralized with Tris buffer. ScFv fragments 14.18 were then transferred to phosphate buffer (PBS) in Amicon Ultra-4 10 kDa centrifugal filters (Merck, Darmstadt, Germany) and sterilized through a 0.22 μm membrane filter. Protein concentration was calculated at a wavelength of 280 nm by BioDrop μLITE spectrophotometer (BioDrop, Cambridge, UK).

### 4.2. Generation of Antibody Fragment Conjugates

For generation of pegylated scFv fragments of antibodies, maleimide-activated polyethylene glycol molecules that selectively react with thiol groups of cysteine were used, namely, monofunctional methoxy PEG maleimides with molecular weights 5 and 10 kDa and Y-shape PEG maleimide with molecular weight 40 kDa (for simplicity, they are abbreviated PEG-maleimide-5, PEG-maleimide-10, and PEG-maleimide-40, respectively), bifunctional PEG dimaleimides with molecular weight 2 kDa and 5 kDa (PEG-di-maleimide-2 and PEG-di-maleimide-5, respectively), and tetrafunctional 4arm PEG maleimide with molecular weight 10 kDa (PEG-tetra-maleimide-10) (all from JenKem Technology, Plano, TX, USA). For C-terminal cysteine reduction, prior to the pegylation reaction, TCEP (tris(2-carboxyethyl)phosphine) from a stock solution of 10 mM to a final concentration of 0.5 mM was added to 2.5 mg/mL scFv fragments in the pegylation buffer (20 mM phosphate buffer supplemented with 50 mM NaCl, 10 mM ethylenediaminetetraacetic acid (EDTA), pH 6.0). The solution was incubated for 60 min with gentle agitation at RT, followed by removal of the reducing agent by centrifugation through Amicon Ultra filters with 10 kDa filtration cutoff or by Zeba Spin Desalting Columns, 7 K molecular weight cut-off (Thermo Fisher Scientific, Waltham, MA, USA). Immediately after that, stock solutions of corresponding PEG-maleimides were added to the antibody fragment solution in different ratios, and the pegylation reaction was carried out for 16–18 h with gentle agitation at 4 °C. Pegylation efficiency was assessed by SDS-PAGE. To separate pegylated fragments from unreacted molecules and to isolate individual pegylated monomers and polymers of scFv fragments 14.18, size-exclusion chromatographic purification was performed (Superdex 200 10/300 GL column (GE Healthcare, Chicago, IL, USA), and PBS eluent sterilized through 0.22 μm membrane filter, flow rate 0.6 mL/min). The resultant pure fractions were concentrated on Amicon Ultra 10 kDa filters and sterilized through a 0.22 μm membrane filter. Protein concentration was determined spectrophotometrically, as described above.

### 4.3. SDS-PAGE and Western Blot Analysis

SDS-PAGE and western blot analysis of non-modified and conjugated scFv fragments were performed as described before [17]. In brief, the samples were resolved in 12% reducing SDS-PAGE, and the resultant gels were either stained with Coomassie R250 or transferred onto nitrocellulose membranes using the Semi-Dry Blotter V10-SDB (Biostep, Burkhardtsdorf, Germany). Membranes were incubated in blocking buffer (5% nonfat dried milk, 0.05% Tween 20 in PBS) for 1 h at RT, followed by incubation with HRP-labelled anti-FLAG antibodies (1:6000) in PBS supplemented with 0.05% Tween 20 (PBS-T) for 1 h at room temperature (RT). Membranes were then rinsed out four times in PBS-T, and the immunoreactive proteins were visualized with 1-Step Ultra TMB (3,3’,5,5’-tetramethylbenzidine) -Blotting Solution (Thermo Fisher Scientific, Waltham, MA, USA) according to the manufacturer’s instructions. Gels and membranes were analyzed in Gel Doc EZ Imager and Image Lab software (Bio-Rad, Hercules, CA, USA).

### 4.4. Enzyme-Linked Immunosorbent Assay (ELISA)

*Direct ELISA.* Nunc MaxiSorp high protein-binding capacity 96 well ELISA plates (Thermo Fisher Scientific, Waltham, MA, USA) were coated with gangliosides GD2, GM2, GD1b, and GD3 at concentration of 0.1 μg in 100 μL of ethanol per well. Gangliosides were obtained according to the method applied in our previous work [38] and kindly provided by Dr. Mikhalyov (Shemyakin-Ovchinnikov Institute of Bioorganic Chemistry, Russian Academy of Sciences, Moscow, Russian Fedration). Following air drying, plate wells were blocked with 100 μL 2% bovine serum albumin (BSA) in PBS-T per well for 2 h at RT. ScFv fragments 14.18 or biotinylated antibodies 14G2a (BioLegend, San Diego, CA, USA) (100 μL per well in PBS-T) were added in triplicates at different concentrations. Following incubation for 1.5 h at RT and washing with PBS-T, HRP-labelled anti-FLAG antibodies or HRP-labelled streptavidin (both 1:6000), respectively, were added to the wells. After 40 min of incubation at RT and further washing, 1-Step Ultra TMB-ELISA Substrate Solution (Thermo Fisher Scientific Waltham, MA, USA) was added to the wells, and the color reaction optical density (OD) was measured at 450 nm by Multiscan FC microplate reader (Thermo Fisher Scientific, Waltham, MA, USA). Percent of cross-reactivity was calculated as ratio of TMB color reaction OD_450_ in GM2-, GD1b-, or GD3-coated wells to OD_450_ in GD2-coated wells. Sub-saturating concentration for biotinylated antibody 14G2a was determined for competitive ELISA.

*Competitive ELISA.* Similarly to direct ELISA, plates were coated with ganglioside GD2 and blocked with 2% BSA in PBS-T. Following this, a mix of constant amount of biotinylated GD2-specific antibodies 14G2a at sub-saturating concentration and scFv fragments at different concentrations was incubated with immobilized ganglioside GD2 for 1.5 h at RT. Plates were then washed with PBS-T, and HRP-labelled streptavidin (1:6000) was added. IC50% values for scFv fragments were determined from an inhibition curve using SigmaPlot software (Systat Software Inc., San Jose, CA, USA).

### 4.5. Cell Lines

GD2-positive mouse lymphoma EL-4 cell line was cultured in Roswell Park Memorial Institute medium (RPMI-1640), human neuroblastoma IMR-32 in Eagle’s Minimum Essential Medium (EMEM), and human neuroblastoma NGP-127 in Dulbecco’s Modified Eagle Medium (DMEM). All culture media were supplemented with 10% heat-inactivated fetal bovine serum, 2 mM *L*-glutamine, 100 μg/mL penicillin, and 100 U/mL of streptomycin (all—Thermo Fisher Scientific, Waltham, MA, USA)). Cell lines were kindly provided by Dr. A. Buzdin (Shemyakin-Ovchinnikov Institute of Bioorganic Chemistry, Russian Academy of Sciences, Moscow, Russian Federation).

### 4.6. Flow Cytometry

Staining of EL-4, IMR-32, and NGP127 cells with scFv fragments 14.18 was performed as described previously [39]. In brief, cells were detached from the culture plates (adherent cells were trypsinized and washed twice in PBS), incubated with scFv fragments 14.18 (1 μg per 10^6^ cells) for 1 h, and then washed in PBS supplemented with 1% FBS and 0.02% sodium azide. Next, the cells were incubated with anti-FLAG antibodies (1:500) for 40 min, and then washed twice in PBS. All procedures were performed at 4 °C. The samples were immediately analyzed using EPICS Coulter XL-MCL flow cytometer (Beckman Coulter, Porterville, CA, USA). In each sample at least 5,000 events were collected. For all samples, the analysis was performed in triplicate. The data were analyzed using FlowJo and WinMDI software.

### 4.7. MTT Assay

Antibody-induced decrease in cell viability was analyzed by colorimetric MTT (3-[[4,5]-dimethylthiazol-2-yl]-2,5-diphenyltetrazolium bromide; purchased from Sigma-Aldrich, USA) assay previously described by Denizot and Lang [40] with modifications described earlier [41]. Briefly, tumor cells were cultured in 96-well flat-bottom tissue culture plates (10^4^ cells/well, Greiner, Austria) with serial dilutions of scFv fragments or full-length antibodies (chimeric 14.18 and mouse 14G2a) for 72 h under standard culture conditions. Following incubation, the MTT solution (final concentration 250 μg/mL) was added to each sample for 4 h. Reaction optical density OD was assessed by Multiscan FC microplate reader at a wavelength of 540 nm. Cell viability was calculated using the formula (OD _treated cells_ − OD _blank_)/(OD _control cells_ − OD _blank_) × 100%, where OD _blank_ represents OD in control wells containing no cells. Dose-response curves were generated using SigmaPlot software (Systat Software Inc., San Jose, CA, USA). All MTT experiments were reproduced at least three times.

### 4.8. Propidium Iodide (PI) Assay

Analysis of cell death as determined by DNA fragmentation was performed using propidium iodide (PI) staining in accordance with the previously described method [42] with modifications [43]. Tumor cells (10^6^ cells per sample) were incubated with scFv fragments or ch14.18 at a concentration of 50 μg/mL, or m14G2a (20 µg/mL) for 24 h under standard culture conditions. Cells were subsequently fixed and permeabilized with ice-cold ethanol for 60 min at 4 °C, and washed twice with PBS by centrifugation for 10 min at 300 *g*. Cell pellets were resuspended in DNA staining buffer (PBS supplemented with 20 μg/mL PI (Sigma-Aldrich), 20 μg/mL RNase A (Thermo Fisher Scientific)) and further incubated for 30 min at RT. For all samples, cell death analysis was performed in triplicate. Percent of hypodiploid cells was evaluated by decreased fluorescence with EPICS Coulter XL-MCL flow cytometer.

### 4.9. Analysis of Circulation Time of Modified scFv Fragments 14.18 in the Blood of Balb/c Mice

On the first stage, naked scFv fragments 14.18 (or chimeric antibodies 14.18) were labelled with the fluorescent dye Sulfo-Cyanine5 maleimide (Sulfo-Cy5-Mal, Lumiprobe, Moscow, Russian Federation) via the C-terminal free cysteine (or via cysteines of interchain disulfide bonds for full-length mAb), whereas pegylated sFv fragment conjugates were labelled with Sulfo-Cyanine5 N-hydroxysuccinimide ester (Sulfo-Cy5-NHS, also from Lumiprobe) via side chain amine groups of lysine. For fluorescent labelling of naked scFv fragments or antibodies, initially, TCEP from a stock solution of 10 mM to a final concentration of 0.5 mM was added to 2.5 mg/mL scFv fragments or antibodies in 20 mM phosphate buffer supplemented with 50 mM NaCl, 10 mM EDTA, pH 6.0, followed by incubation of the reaction mix for 60 min with gentle agitation at RT. The reducing agent was removed by centrifugation on a Zeba Spin Desalting Column, and Sulfo-Cy5-Mal was added to the protein solution in a molar excess of 5:1 (2:1 for full-length mAb). After 2 h incubation on an orbital shaker at RT, unbound dye was removed by a Zeba Spin Desalting Column.

For fluorescent labelling of pegylated scFv fragment conjugates, Sulfo-Cy5-NHS dye was added in different molar ratios to 2 mg/mL antibody fragment conjugate solutions in PBS. Both the labelling reaction and the subsequent removal of unbound dye were performed as described above. Dye molecule/protein molecule ratio within the generated Sulfo-Cy5-labelled pegylated scFv fragment conjugates was in the range of 0.63–0.68, as calculated by BioDrop μLITE spectrophotometer using the formula $\frac{N_{fluor}}{N_{mAb}}=\frac{A_{fluor}\times \varepsilon_{mAb}}{\left(A_{mAb}-A_{fluor}\times CF\right)\times \varepsilon_{fluor}}$, where $\varepsilon_{fluor}$= 271,000 L⋅mol^−1^⋅cm^−1^, $\varepsilon_{mAb}$= 38720 L⋅mol^−1^⋅cm^−1^ for scFv fragments and 210000 L⋅mol^−1^⋅cm^−1^ for IgG, CF_280_ = 0.04.

On the second stage, fluorescently labelled antibody fragment conjugates (100–150 μL) were intravenously injected into C57BL/6 mice (Central Farm, Moscow region, Russian Federation) (*n* = 3 for each conjugate) in the amount of 150 μg / mouse (7.5 mg / kg). All experiments with mice were approved by the Institutional Animal Commission protocol # 259 from 05.15.2018. Blood aliquots (30 μL) were collected via tail vein at certain time intervals after administration of scFv antibody fragment conjugates, and the combined blood from three animals in each group was collected in heparin tubes. Blood plasma was obtained by centrifugation at 10,000 *g* for 10 min. Blood plasma from intact animals served as autofluorescence reference. Fluorescence intensity measurements were performed on a GloMax-Multi Detection System plate fluorometer (Promega, Madison, WI, USA) with the excitation filter at 625 nm and the absorbance filter at 660–720 nm. The ratios of mean fluorescence intensity of Sulfo-Cy5-labelled scFv fragments in blood plasma of experimental animals to the mean fluorescence intensity of blood plasma of intact mice (blood-to-background (B-T-B) ratio) over time were calculated.

### 4.10. Tumor Uptake of Sulfo-Cy5-Labelled scFv Fragments 14.18 in EL-4 Syngeneic Tumor Model

All experiments with mice were approved by the Institutional Animal Commission protocol # 259 from 05.15.2018. Subcutaneous EL-4 tumor implants were established in 12- to 14-week-old syngeneic C57BL/6 mice (Central Farm, Moscow region, Russia) by s.c. inoculation of 5 × 10^6^ EL-4 cells in the right flank. Once tumors reached 300 mm^3^, mice were divided randomly into the control and experimental groups (*n* = 3 for each group). The experimental groups received i.v. injection of 150 μg per mouse (7.5 μg/kg) Sulfo-Cy5-labelled scFv fragments, Sulfo-Cy5-labelled pegylated tetra-scFv fragments, or Sulfo-Cy5-labelled antibodies 14.18, whereas the control group received intravenous (i.v.) injection of PBS. Mice were euthanized 2 h or 24 h post-injection, and tumors were collected and processed for fluorescence measurements, as described in [44]. In brief, tumors were weighed and homogenized in 1 mL PBS supplemented with Triton X100 (0.1%) per 200 mg of tissue. Aliquots were diluted with an equal volume of acid-ethanol solution (70% EtOH, 12.5 mL HCl in H_2_O) and incubated at 4 °C overnight. Then, samples were cleared by centrifugation for 20 min at 15,000 rpm, followed by transfer of 200 μL supernatant from tumors to 96-well plates (Corning, Durham, NC, USA) and fluorescence measurement as described above. Mice from the control group served as an autofluorescence reference for the calculation of tumor-to-background ratio (T-T-B).

### 4.11. Statistical Analysis

Graphs were created using SigmaPlot and MS Excel software. The data are represented as mean ± standard error of the mean (SEM) of at least three independent experiments, or as one representative experiment from three. Statistical analysis was performed using Student’s t-test. Significance levels of *p* < 0.05 were considered statistically reliable.

## 5. Conclusions

The findings of this study provide the rationale for improving therapeutic characteristics of GD2-specific antibody fragments by multimerization and propose a strategy to generate such molecules. Multimeric antibody fragments may be employed as targeting moieties within diagnostic and therapeutic drugs. On their basis, bispecific antibody fragments and conjugates with drugs or radioactive isotopes may be developed that will possess improved pharmacokinetic and pharmacodynamic properties and reduced side effects due to the absence of the Fc antibody fragment.

## Supplementary Materials

The following are available online at https://www.mdpi.com/1420-3049/24/21/3835/s1. Figure S1: Western blot of the pegylation reaction products; membrane was incubated with HRP-labelled anti-FLAG antibodies (1:6000); 1, pegylation with PEG-di-maleimide 5 kD; 2, intact scFv fragments 14.18; 3, pegylation with PEG-tetra-maleimide 10 kD. Values from densitometry analysis of western blot bands in lanes 1 and 3 were normalized by intensity of the intact scFv fragment (value for intact scFv from each individual lane set to 1.0). Blot images were acquired and analyzed in Bio Rad Gel Doc EZ Imager and Image Lab 6.0.1 Software (BioRad, USA). Figure S2: western blot following size-exclusion chromatographic purification, pegylation reaction with PEG-di-maleimide 5 kD; 1, fraction of pegylated di-scFv fragments; 2, fraction of pegylated mono-scFv fragments; 3, fraction of non-pegylated scFv fragments; 4, total protein mixture after pegylation with PEG-di-maleimide 5 kD. Values from densitometry analysis of western blot bands in lanes 2, 3, and 4 were normalized by intensity of the intact scFv fragment (value for intact scFv from each individual lane set to 1.0). Values in lane 1 were normalized by intensity of monovalent conjugate of scFv. Blot images were acquired and analyzed in Bio Rad Gel Doc EZ Imager and Image Lab 6.0.1 Software (BioRad, USA). Figure S3. Direct cell death effects following incubation with intact scFv fragments, pegylated monomers of scFv fragments, pegylated di- and tetra-scFv fragments, and full-length GD2-specific antibodies (chimeric 14.18 and mouse 14G2a). A—GD2-positive EL-4 cell line, MTT-assay after 72 h incubation; B—EL-4 cell line, PI-test after 24 h incubation. Control cells incubated with PBS. Data are represented as mean ± SEM. *—indicates a value that significantly differs from the control value at *p* < 0.05 (Student’s t-test, *n* = 5). Figure S4. Accumulation of Cy5-labelled intact scFv and pegylated tetra-scFv fragments in the organs of C57BL/6 mice 24 h post-injection.
