# Supplementary material for: Multimerization through Pegylation Improves Pharmacokinetic Properties of scFv Fragments of GD2-Specific Antibodies
Source: Molecules. 2019 Oct 24;24(21):3835. doi: 10.3390/molecules24213835 (PMC6864547; doi:10.3390/molecules24213835)
Supplement: Supplementary file 1 [file molecules-24-03835-s001.pdf]

Supplementary Materials

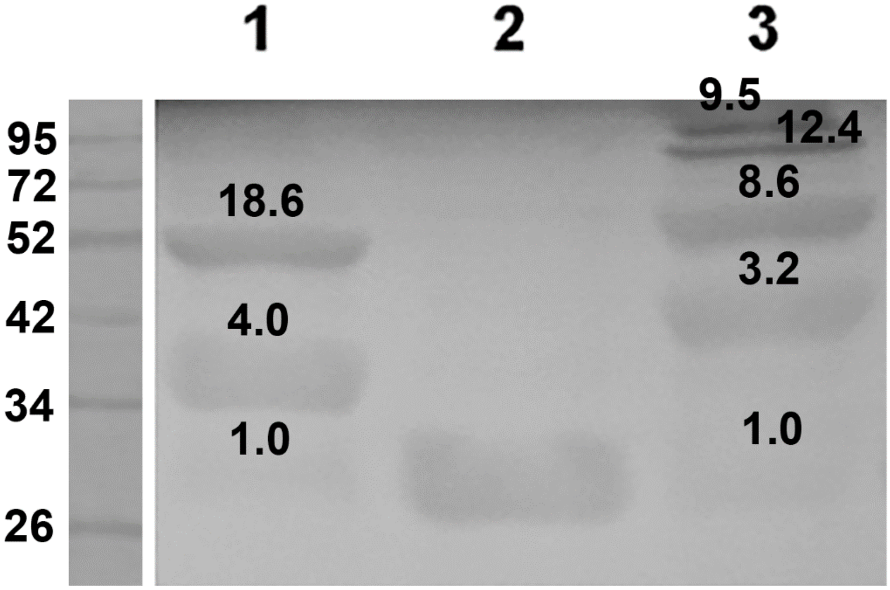

Figure 1. Western blot of the pegylation reaction products; membrane was incubated with HRP-labelled anti-FLAG antibodies (1:6000); 1, pegylation with PEG-di-maleimide 5 kD; 2, intact scFv fragments 14.18; 3, pegylation with PEG-tetra-maleimide 10 kD. Values from densitometry analysis of western blot bands in lanes 1 and 3 were normalized by intensity of the intact scFv fragment (value for intact scFv from each individual lane set to 1.0). Blot images were acquired and analyzed in Bio Rad Gel Doc EZ Imager and Image Lab 6.0.1 Software (BioRad, USA).

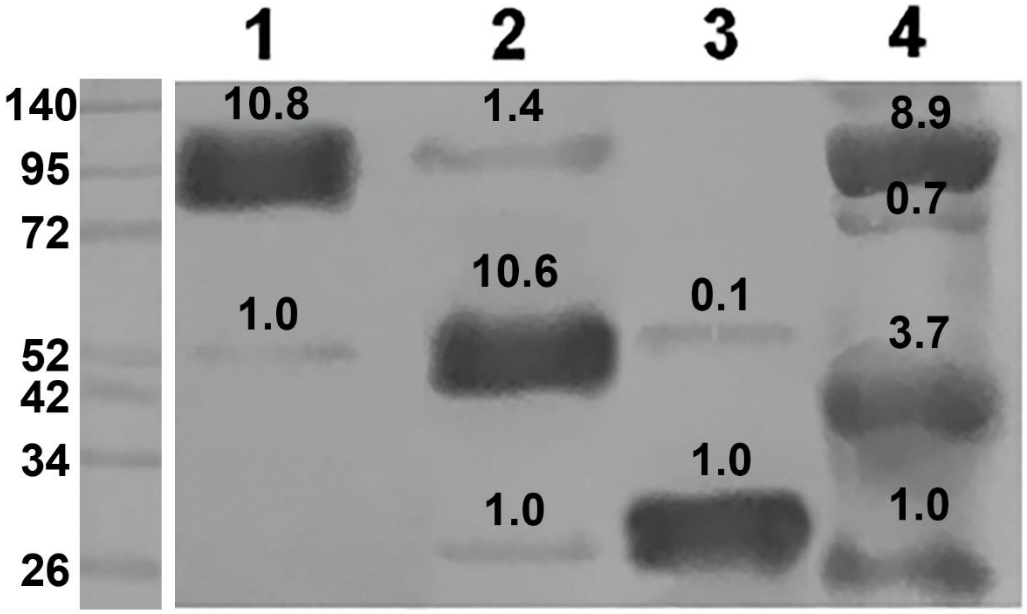

Figure 2. Western blot following size-exclusion chromatographic purification, pegylation reaction with PEG-di-maleimide 5 kD; 1, fraction of pegylated di-scFv fragments; 2, fraction of pegylated mono-scFv fragments; 3, fraction of non-pegylated scFv fragments; 4, total protein mixture after pegylation with PEG-di-maleimide 5 kD. Values from densitometry analysis of western blot bands in lanes 2, 3, and 4 were normalized by intensity of the intact scFv fragment (value for intact scFv from

each individual lane set to 1.0). Values in lane 1 were normalized by intensity of monovalent conjugate of scFv. Blot images were acquired and analyzed in Bio Rad Gel Doc EZ Imager and Image Lab 6.0.1 Software (BioRad, USA).

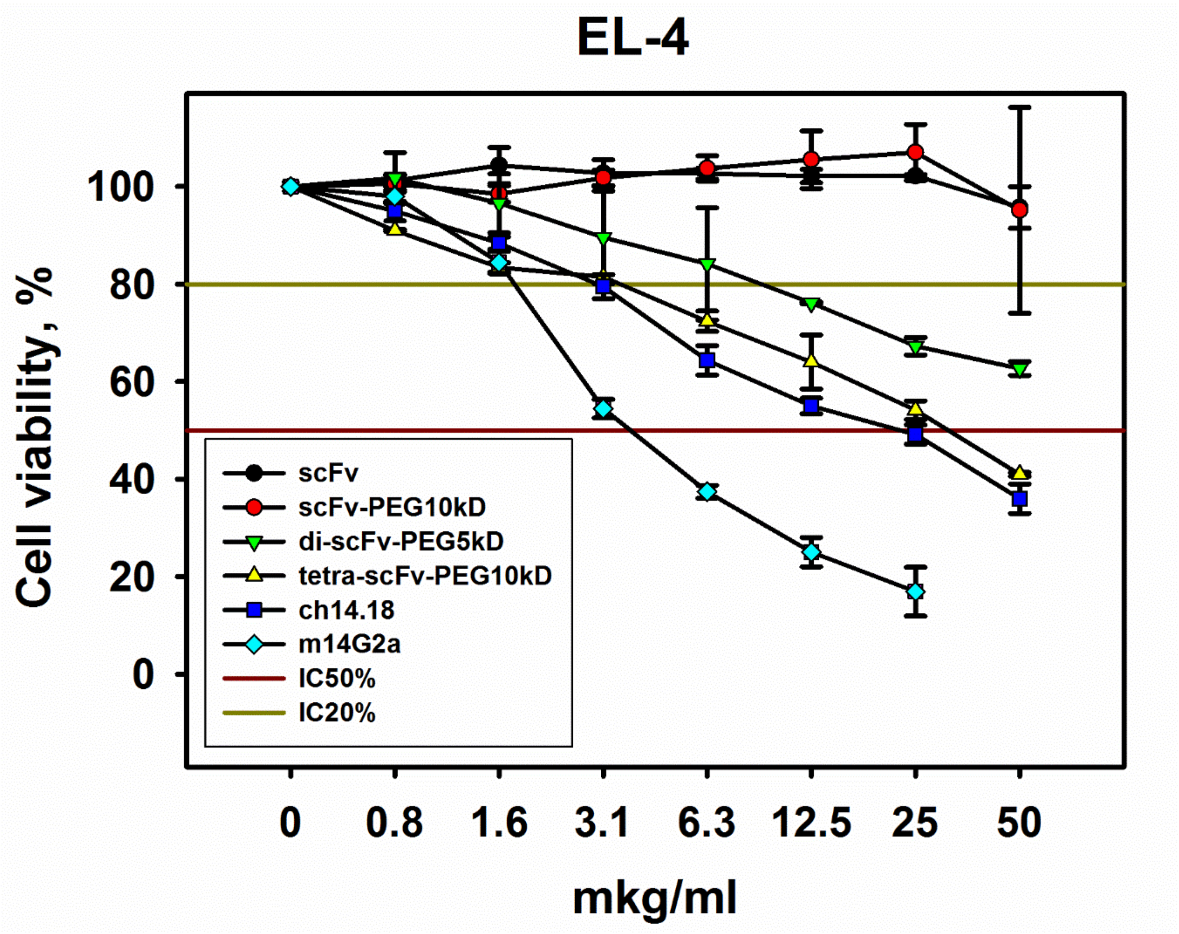

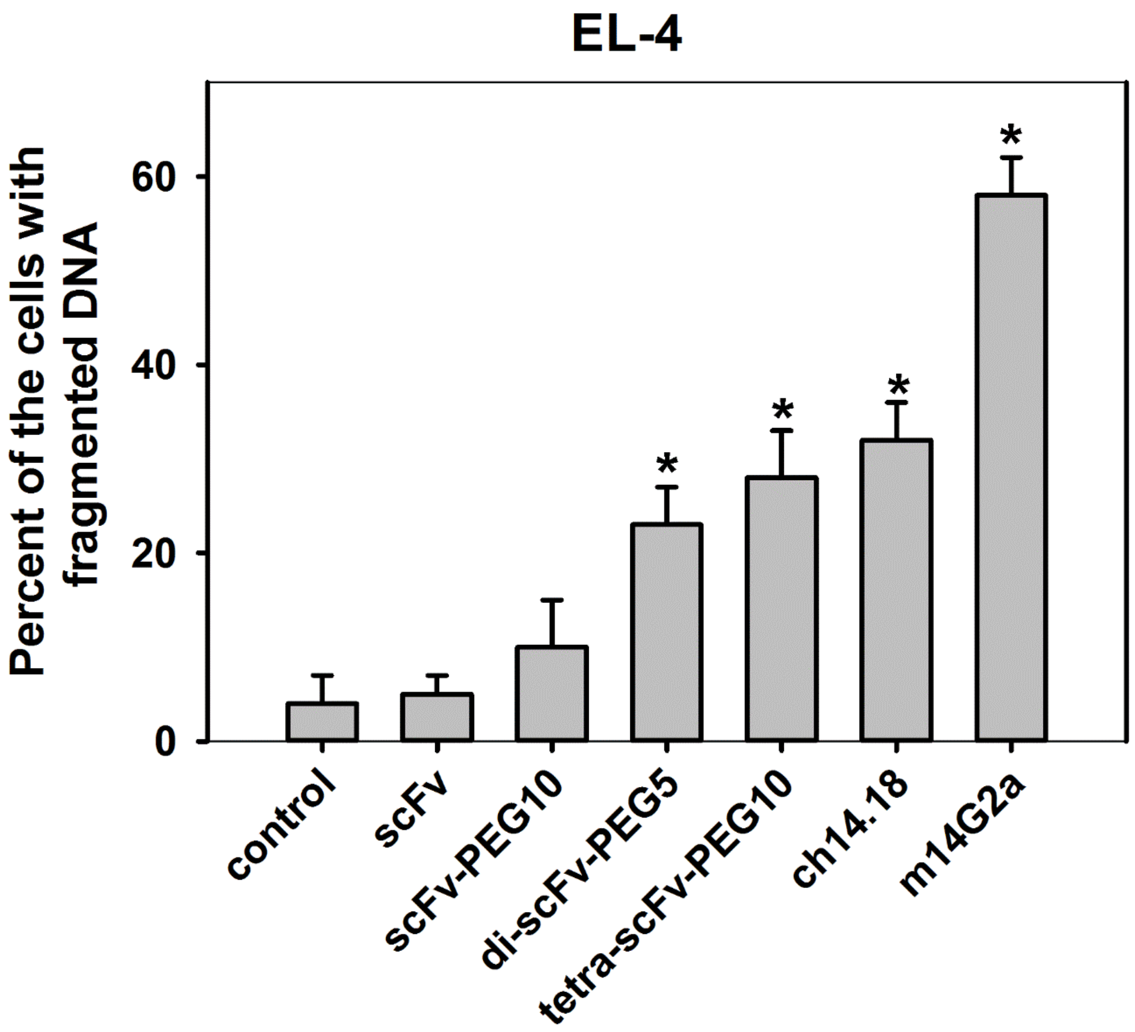

Figure 3. Direct cell death effects following incubation with intact scFv fragments, pegylated monomers of scFv fragments, pegylated di- and tetra-scFv fragments, and full-length GD2-specific antibodies (chimeric 14.18 and mouse 14G2a). A—GD2-positive EL-4 cell line, MTT-assay after 72 h incubation; B—EL-4 cell line, PI-test after 24 h incubation. Control cells incubated with PBS. Data are represented as mean ± SEM. *—indicates a value that significantly differs from the control value at $p < 0.05$ (Student’s t-test, $n = 5$).

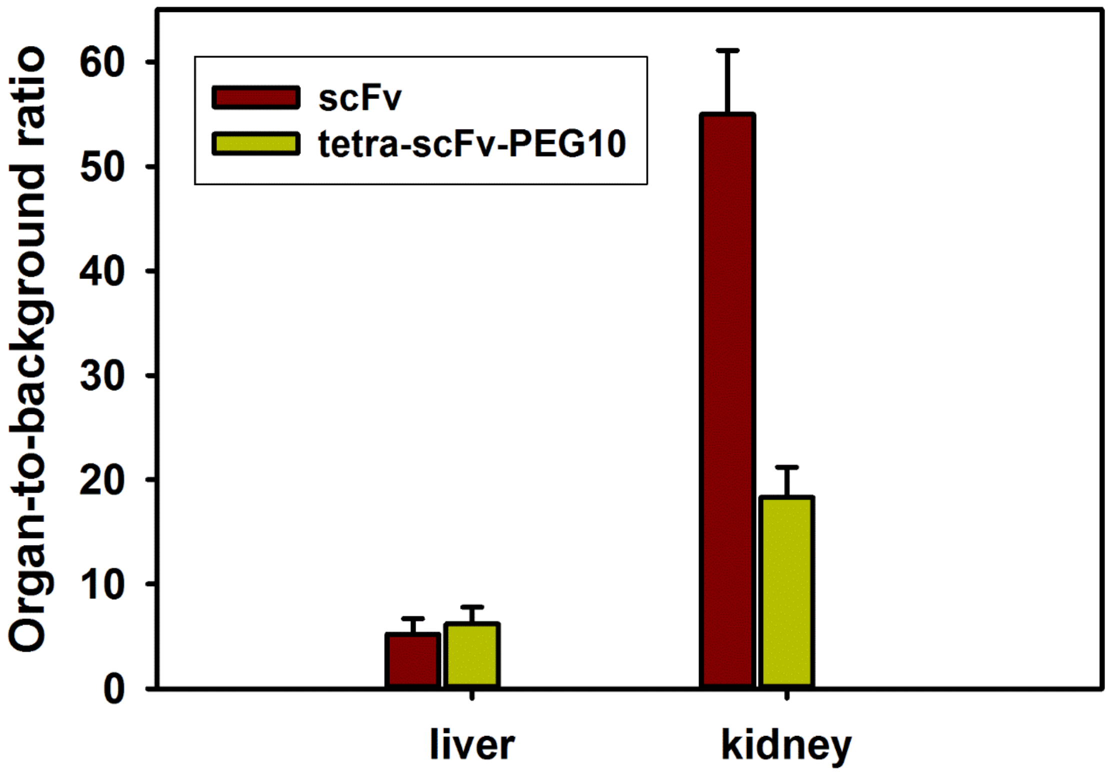

Figure 4. Accumulation of Cy5-labelled intact scFv and pegylated tetra-scFv fragments in the organs of C57BL/6 mice 24 h post-injection.
